# A Markov Chain Model for Mental Health Interventions

**DOI:** 10.3390/ijerph20043525

**Published:** 2023-02-16

**Authors:** David Claudio, Sally Moyce, Tyler Albano, Ekeoma Ibe, Nick Miller, Marshall O’Leary

**Affiliations:** 1 Department of Mechanical and Industrial Engineering, University of Massachusetts Lowell, Lowell, MA 01854, USA; 2 College of Nursing, Montana State University, Bozeman, MT 59717, USA

**Keywords:** Markov chains, absorbing chains, mental health

## Abstract

Poor mental health affects nearly one billion people worldwide and can end in suicide if not treated. Unfortunately, stigma and a lack of mental healthcare providers are barriers to receiving needed care. We developed a Markov chain model to determine whether decreasing stigma or increasing available resources improves mental health outcomes. We mapped potential steps in the mental health care continuum with two discrete outcomes: getting better or committing suicide. Using a Markov chain model, we calculated probabilities of each outcome based on projected increases in seeking help or availability of professional resources. Modeling for a 12% increase in awareness of mental health concerns yielded a 0.39% reduction in suicide. A 12% increase in access to professional help yielded a 0.47% reduction in suicide rate. Our results show that expanding access to professional services has a higher impact on reducing suicide rates than creating awareness. Any intervention towards awareness or access positively impacts reducing suicide rates. However, increased access results in a higher reduction in suicide rates. We have made progress in increasing awareness. Awareness campaigns help to increase recognition of mental health needs. However, focusing efforts on increasing access to care may have a higher impact on reducing suicide rates.

## 1. Introduction

The World Health Organization (WHO) reported in 2020 that mental disorders affect nearly one billion people worldwide [1]. According to the same report, one person dies every 40 s by suicide [1]. The situation has been exacerbated by the COVID-19 pandemic [2,3,4].

Many issues can lead to mental disorders, including unemployment, poverty, financial struggles, alcohol or drug abuse, homelessness, uncertainty, isolation, fear, and large-scale movement restrictions [3,5,6]. An additional problem is the reluctance of people to seek help when they start experiencing emotional distress, anxiety, or depression [7,8,9,10,11]. Psychiatric intervention for individuals with worsening mental health is critical to treating depression and anxiety and preventing suicide [12,13,14].

Two major barriers prevent persons with poor mental health from seeking professional care: stigma and access. Stigma is a complex concept that occurs when an individual deems the need for mental health treatment shameful [7]. Reluctance to seek formal and informal support is associated with self-stigma [7,8,10]. There is a sense of shame that accompanies self-stigma, and the adaptive response to this shame is secrecy; this results in not acknowledging or disclosing mental health problems and not seeking treatment. Access to mental health services is another barrier due to an inability to find a provider or to the high cost of available care [15]. For example, much of the population in the United States live in a mental health provider shortage area [16]. 

In response to the disparities in access to care, recent studies have focused on mental health prevention and treatment programs [8,9,10,11,12,13,14]. Researchers have used Markov chain models to analyze and evaluate mental health programs in the past, including suicide prevention models. For example, Yip et al. [17] estimated population suicide risk as a dynamic system to evaluate the effectiveness of suicide prevention programs. They assumed the population remained in equilibrium as births replaced the deceased. While this model was useful for studying the effectiveness of suicide prevention programs, we focused our model on the individual. We aimed to elaborate on the choices individuals can make to determine their state in a Markov chain.

Oskooyee et al. [12] used real clinical data to model depression severity through Markov transitional matrices. Their Markov chain contains probabilities of moving from one state of depression to another, where state 0 is no depression and state 3 is severe depression [12]. Through their case study, they demonstrated the effectiveness of professional help with real measured data. 

Our research study developed a Markov chain model that details the complicated web an individual enters when dealing with any mental illness (AMI). AMI is defined as a mental, behavioral, or emotional disorder that can range from no impairment to mild, moderate, and even severe impairment [18]. We developed a transitional matrix that defines the probability of jumping from one attitude or mental state in the Markov chain to another. Throughout the article, we will be answering two main questions: 

Q1: What are the probabilities of moving from one mental state to another within our proposed Markov chain?

Q2: Where would the allocation of resources have the most positive impact, increasing awareness (decreasing stigma) so that an individual seeks help, or increasing access to professional resources? 

To answer the first question, we performed a literature review to accurately map the cascade effect these probabilities have on the different states within the Markov chain. Consequently, through multiple interpolations, we explored whether resources would be better spent by strengthening the system, which is actively supporting people looking for help, or by increasing the ease of access and awareness of such resources. 

## 2. Methods

**Figure 1:**
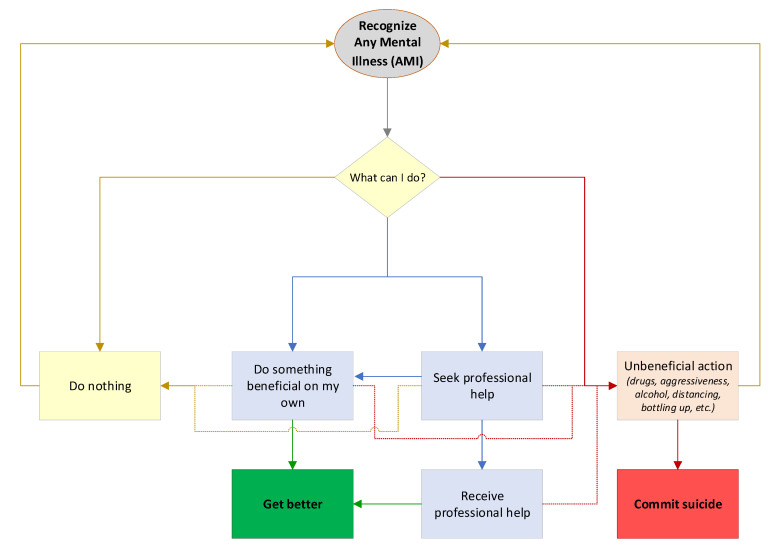
Flowchart describing how the actions of an individual determine the potential outcomes.

Different stages of the potential actions of a person with AMI were mapped through a community-based participatory research process [4,19,20]. Members of a community advisory board seeking to reduce poor mental health outcomes created a flowchart to illustrate various stages in the experience of a person with AMI. Figure 1 presents the flowchart as a person moves through recognition of AMI to one of four states: (1) complacency, (2) attempting to remedy the issue alone through healthy behavior, (3) seeking professional help, or (4) electing to participate in unhealthy behavior. For this research, we considered each occurrence in the flowchart as independent of past and future occurrences. In reality, mental health is a constantly evolving state of mind, and an individual may progress through the flowchart many times. We assumed that the end goal was to feel relief from AMI, and we created two discrete outcomes: get better or commit suicide.

The flowchart served as a baseline to develop our Markov chain model. A couple of assumptions were made to create the initial Markov chain model. Firstly, the model assumes that doing something beneficial and seeking professional help are mutually exclusive. Individuals could do something beneficial as part of their treatment, but the model differentiates them based on doing something as an individual versus doing something at the recommendation of a professional. Furthermore, actions and individual attempts that are not successful at relieving AMI were not included. For simplicity’s sake, we modeled this as going straight to the final negative outcome (suicide).

**Figure 2:**
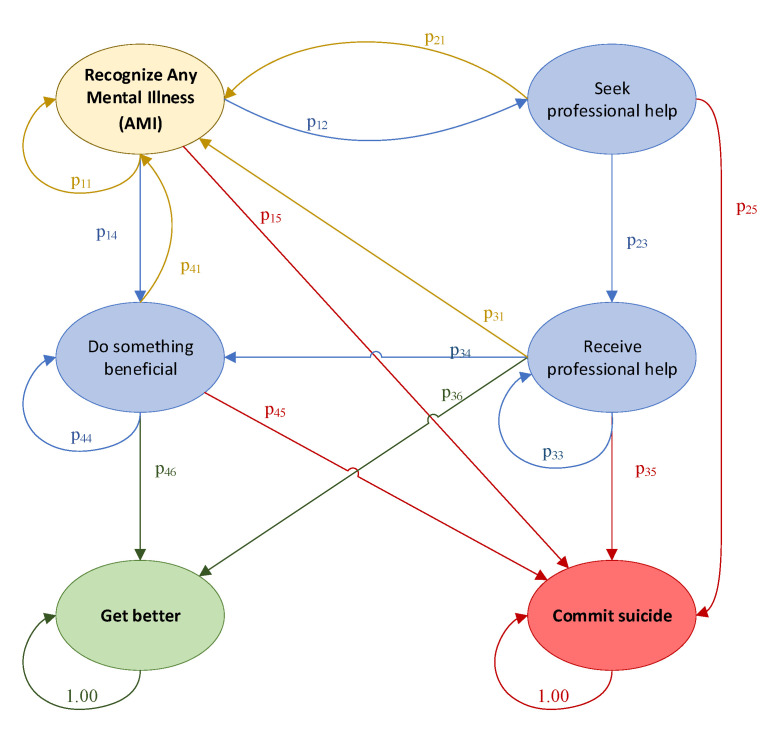
Markov chain for any mental illness with two absorbing states: get better and commit suicide.

**Figure 3:**
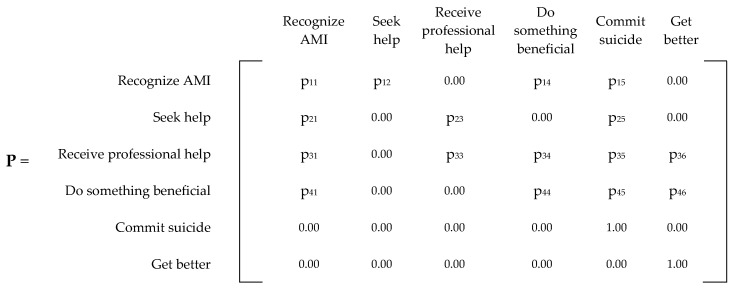
Generalized P matrix for any mental illness (AMI).

Figure 2 displays the generalized Markov chain model for AMI, while Figure 3 displays the generalized transitional matrix of the Markov chain (denoted by **P**). Each probability, p_ij_, represents the probability of transitioning from state i to state j, given that an individual is in state i. One of the most basic assumptions of Markov chains is that the future depends only on the state we are in (current state), not on how we arrived at this state. In other words, the future depends on the present and not on the past [21]. 

Since we looked at each mental health occurrence independently of past and future occurrences, the “get better” state was modeled as an absorbing state. An absorbing state is one which, once entered, cannot be left [21]. For obvious reasons, the “commit suicide” state was also modeled as an absorbing state.

**Figure 4:**
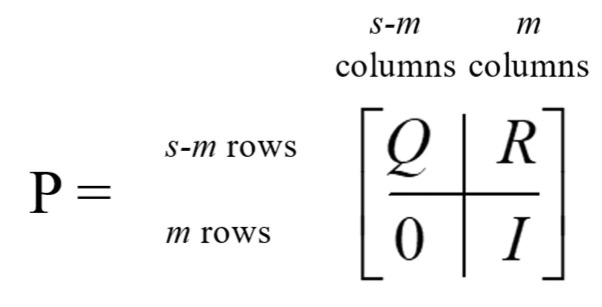
Canonical form of an absorbing Markov chain probability matrix [21].

An absorbing Markov chain with *s* number of states can be represented by an *s × s* probability matrix, denoted by **P**. The canonical form of an absorbing matrix with *m* absorbing states can be represented with four matrices within **P**. Figure 4 displays the canonical form of an absorbing Markov chain probability matrix [21]. The matrix denoted by **Q** represents the probability of transitioning from a transient state to another, while the **R** matrix describes the probability of transitioning from a transient state to an absorbing state. The **0** matrix contains only zeroes (0), while **I** represents an *m × m* identity matrix created by grouping the absorbing states.

The matrix **(I-Q)^−1^** is referred to as the Markov chain’s fundamental matrix, which provides the expected number of visits from one transient state to another before being absorbed. The matrix **(I-Q)^−1^ R** provides the probability of eventually ending in an absorbing state from any transient state.

The first task was to answer research question #1 by conducting a literature review to accurately map the probabilities within our Markov chain model. A total of sixteen probabilities needed to be populated (p_11_, p_12_, p_14_, p_15_, p_21_, p_23_, p_25_, p_31_, p_33_, p_34_, p_35_, p_36_, p_41_, p_44_, p_45_, and p_46_). With these probabilities, we created our current state model to serve as a baseline. 

We then conducted a series of experiments in which we increased two factors: awareness of seeking professional help (increased awareness) and access to receiving professional help (increased access). Each factor was increased by 4, 8, and 12% from the current state while the other factors were left constant. We then conducted a third experiment in which both factors were increased 2, 4, and 6% concurrently (which corresponds to a combined increase of 4, 8, and 12%). In all three experiments, we were interested in reducing the overall probability that someone recognizing AMI would eventually commit suicide. With these experiments, we wanted to explore whether resources would be better spent by increasing awareness (p_12_) or strengthening the system to increase access for those seeking professional help (p_23_).

## 3. Results

### 3.1. Current State

The review of the literature revealed that the global suicide rate is approximately 1.4% in the general population, but among people with AMI, the rate jumps to 6.5% [22]. We used the 1.4% rate as the probability of moving from any state into the “commit suicide” state (p_15_ = p_25_ = p_35_ = p_45_ = 0.014). As previously stated, suicide is an absorbing state and, therefore, p_55_ = 1. We used the 6.5% rate to validate our model with the probability that someone who recognizes any mental illness will eventually commit suicide. 

In the U.S., 45% of individuals with a clinical-level mental problem do not seek professional help [15]. Furthermore, approximately 29% of those individuals who do not seek professional help prefer to manage their challenges on their own or do not think they need mental health treatment or therapy [15]. This led to setting p_14_ = 0.13 (29% of the 45%) and p_11_ = 0.32 (45 − 13%). Consequently, the probability of recognizing AMI and seeking help (p_12_) was estimated as 1 − p_11_ − p_14_ − p_15_ = 1 − 0.32 − 0.13 − 0.014 = 0.536.

Approximately 27.5% of the people not receiving help claim the primary reason as lack of affordability or access [15]. This led to setting p_21_ = 0.124 (27.5% of 45%). This probability represents the chances of ending in state (1,1) given that there is no access or people cannot afford professional help. 

**Figure 5:**
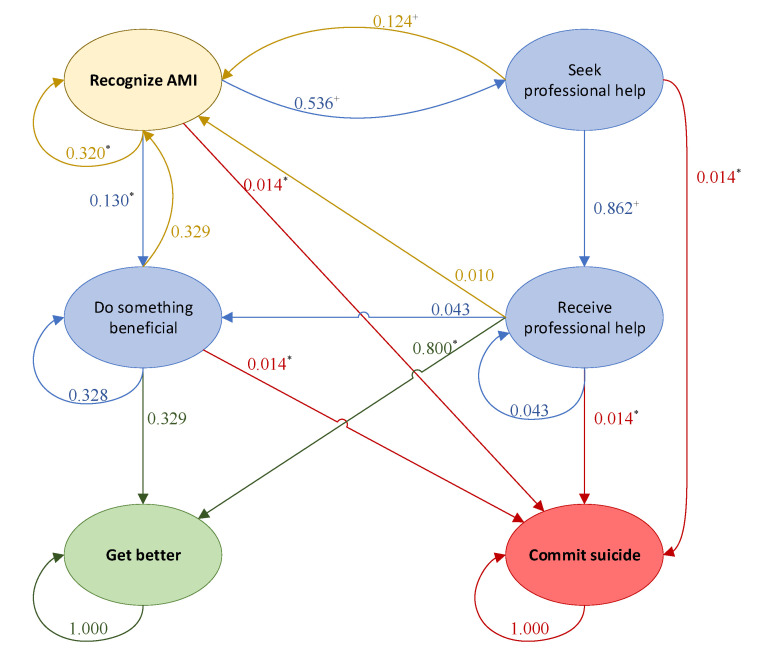
Current state Markov chain model for any mental illness. * Statistic obtained directly from the literature; ^+^ Calculated from statistics obtained from the literature.

In 2020, only 46.2% of adults with AMI in the U.S. received mental health services [18]; alternatively stated, of the 53.6% of people who seek care, 86.2% successfully accessed it. Therefore, we estimated p_23_ = 0.862 to achieve the 46.2% of adults who seek and receive care [18]. Finally, professional help has an estimated 80% effectiveness rate [23], and thus p_36_ = 0.800. Figure 5 presents the current state Markov chain model with the probabilities found in the literature.

Some probabilities within our Markov chain lacked current literature and were missing proper data (p_31_, p_33_, p_34_, p_41_, p_44_, p_46_). They can be classified into one of two major categories: (1) statistics of people who receive professional service with no effect, and (2) statistics regarding what happens to people who decide to do something beneficial on their own. Therefore, these values were inferred and adjusted until we achieved a final probability close to the 6.5% suicide rate for people with mental health problems.

For the probabilities regarding ineffective professional services (p_31_, p_33_, p_34_), we assumed 10% of the population returns to the initial state of realizing they have AMI (p_31_ = 0.100). After removing the chance of committing suicide (0.014), the remaining probabilities were equally divided among the two states (p_33_ = p_34_ = 0.043).

**Figure 6:**
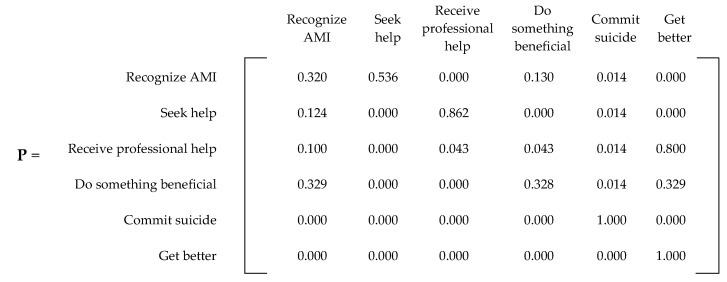
Current state **P** matrix.

**Figure 7:**
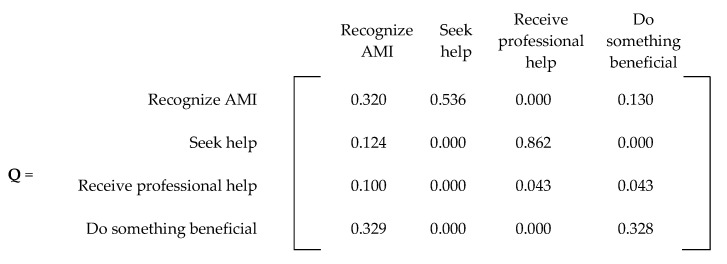
**Q** matrix (current state).

**Figure 8:**
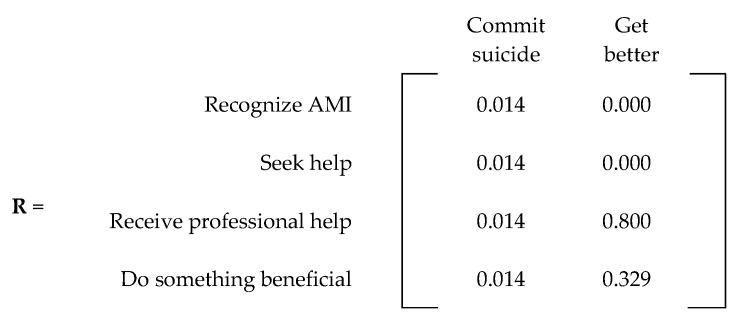
**R** matrix (current state).

For the probabilities regarding doing something beneficial (p_41_, p_44_, p_46_), we assumed that after removing the chance of committing suicide (0.014), the remaining states had an equal chance of being achieved. Therefore, 0.986 was equally divided among the three probabilities (p_41_ = p_44_ = p_46_ = 0.32866). For simplicity, we assigned two probabilities at 0.329 and the third at 0.328. All these probabilities resulted in the **P** matrix presented in Figure 6. For convenience, Figure 7 and Figure 8 present the **Q** and **R** transient and absorbing state matrices for the current state.

**Figure 9:**
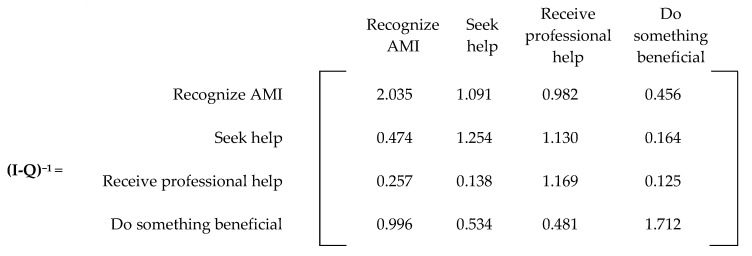
Fundamental matrix (current state).

The fundamental matrix for the current state is found by subtracting the transient matrix from the identity matrix and taking the inverse of the resulting matrix (Figure 9). As seen in Figure 9, the highest number in the matrix is in recognizing AMI. This number (2.035) means an individual will end up in this state twice before getting better or committing suicide.

**Figure 10:**
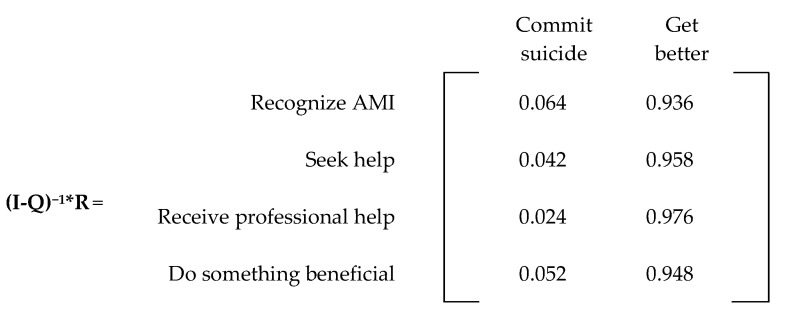
Absorbing matrix (current state).

The absorbing matrix for the current state is found by multiplying the fundamental matrix by the R matrix (Figure 10). From the absorbing matrix, we note a probability of 6.39% that an individual who recognizes AMI will eventually commit suicide, which is close to the 6.5% found in the literature [22]. 

### 3.2. Future State #1: Increasing Awareness of Seeking Professional Help

Increasing awareness of seeking professional help involves increasing the probability of moving from recognizing AMI to seeking professional help (p_12_). Increasing p_12_ results in a decrease in p_11_ and p_14_. The changes on p_11_ and p_14_ were estimated in the same manner as the current state. For example, a 4% increase in seeking professional help results in 41% of the population not seeking help (4% lower than the current state, which was 45%). We continued to assume that 29% of individuals who do not seek professional help prefer to manage their challenges on their own [15]. This results in p_14_ = 11.89% (29% of 41%) and the remainder going to p_11_ (41% − 11.89% = 29.11%).

Table 1 presents the new probabilities according to each scenario. Table 2 presents the probabilities that someone who recognizes AMI will eventually commit suicide for each of the proposed scenarios. The table also presents the suicide rate reduction of each scenario against the current state (6.39%) and the additional rate reduction compared to the previous scenario. 

For example, increasing awareness by 4% from the current state reduces the suicide rate by 0.1419%, whereas increasing awareness from 4% to 8% awareness sees an additional reduction of 0.1278%. The results from Table 2 show that the suicide rate reduces with every scenario, but the reduction slightly weakens as awareness increases. 

### 3.3. Future State #2: Increasing Access to Professional Services

Increasing access to services involves increasing the probability of receiving help for those who seek help (p_23_). Increasing p_23_ only affects p_21_. Table 3 presents the new probabilities according to each scenario, whereas Table 4 presents the results of these experiments. Similar to the previous experiment, the results from Table 4 show that the suicide rate reduces with every scenario, but the reduction weakens as access increases. 

### 3.4. Future State #3: Increasing Awareness and Access to Professional Services

Increasing awareness and access to professional services involves all the probabilities that changed during the previous two experiments (p_11_, p_12_, p_14_, p_21_, and p_23_). We decided to increase both factors by 2, 4, and 6%, which corresponds to a combined increase of 4, 8, and 12%. Table 5 presents the revised probabilities for increasing awareness and access to professional services, whereas Table 6 presents the results of increasing awareness and access concurrently in equal proportions.

## 4. Discussion

While we found relevant statistics related to AMI, suicide rates, and the effectiveness of professional service, our review of the literature revealed a lack of relevant statistics. These statistics can be classified into two major categories: (1) statistics related to ineffective professional services (p_3j_), and (2) statistics regarding people who decide to do something beneficial on their own (p_4j_). For research purposes, these values were estimated and adjusted until we achieved a final probability close to the 6.5% suicide rate for people with AMI. A total of six probabilities in our chain fell into these two classifications, which resulted in the biggest limitation of this study.

**Figure 11:**
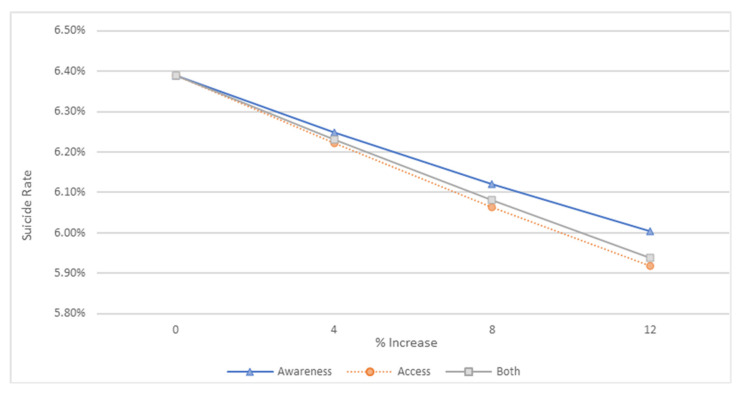
Reductions in suicide rates when increasing awareness, access, and both by 4, 8, and 12%.

We conducted a series of experiments in which we increased two factors: increased awareness and increased access. While the results suggest that increasing either of these factors reduced suicide rates, we found that increasing access resulted in a larger reduction. Interestingly, this reduction was larger than the reduction sought when we combined awareness and access. Figure 11 shows the reductions in suicide rates for each of the three options at different increment levels. 

**Figure 12:**
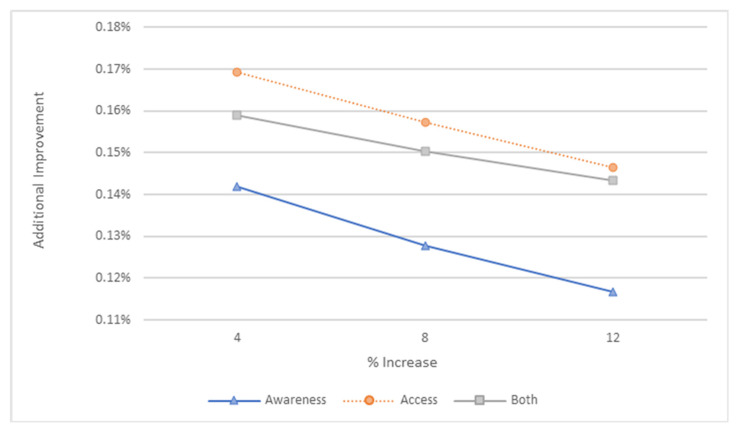
Additional improvements in suicide rates for every 4% increase in awareness and/or access.

With every 4% increase in awareness and/or access, the reduction in suicide rates weakened. Figure 12 shows that increases in awareness result in more diminishing effects than increases in access or increases in both. 

We believe that increased access to health services resulted in a greater reduction in suicide rates, because access eliminates some of the pathways in the model that lead to “commit suicide.” For example, an increase in p_23_, results in a reduction in p_21_; therefore, the chances of going through p_21_ → p_15_ are reduced. There is also a reduction in the chances of taking the p_21_ → p_14_ → p_45_ pathway. Furthermore, assuming an 80% effectiveness of treatment (as found in the literature), increased access would logically result in improvements in AMI. Conversely, increasing awareness without increasing access adds more people to the p_12_ → p_23_ → p_36_ pathway, but not at the same rate as the previous alternative. In addition, it also adds more people to the p_12_ → p_21_ pathway, which could still lead to the previously mentioned pathways of p_21_ → p_15_ and p_21_ → p_14_ → p_45_.

In contrast, the potential effects of increased awareness could be hindered by the percentage that returns to state one (through the p_12_ → p_21_ pathway). In practice, this finding means that increased awareness can only have a positive outcome if people can find the services they are looking for (access). If we increase awareness without increasing access (via affordability or extra capacity), then it is conceivable that a percentage of the population will loop back to the initial state (state 1). The results also show that when we increased access concurrently with awareness at the same rate, the suicide rate reduced at a steady pace. Studies of attitudes toward seeking professional help reveal that many people who suffer from AMI are confident that they will get better on their own or that their symptoms will eventually disappear [24]. Others report concerns that seeking professional help will not make a difference [25]. Therefore, while our results suggest moderate improvement in suicide rates when increasing both awareness and access, other literature suggests interventions that address both aspects may increase mental health professional service utilization overall. Our model did not examine attitudes toward seeking professional help.

These results mathematically display the importance of access to professional services. They also demonstrate the importance of thinking about the benefits of potential intervention plans holistically. For example, rather than spending all our efforts on creating awareness (which might be easier to do than expanding access or improving services), we should perhaps consider spending more than half of our efforts on expanding access for those looking for professional services. Some recommendations for interventions to give people better access to professional help are revoking insurance requirements, consistent online support group attendance, and telehealth recommendations. When our results are extrapolated to the estimated 1 billion persons worldwide with AMI [1], increasing awareness and access could result in 1.42 to 4.73 million suicides that could be prevented each year. 

## 5. Conclusions and Future Recommendations

This research study developed a Markov chain model that details the complicated web people enter when dealing with AMI. Our literature review found reliable statistics on some aspects related to AMI and suicide, but a lack of statistics related to ineffective professional service and the potential benefits of independent actions taken by those with AMI. We also conducted a series of experiments in which we increased two factors, awareness and access. The results reveal that while intervention does reduce suicide rates, access results in a greater reduction. Strategies aimed at improving access, such as telehealth or financial assistance, could result in reduced suicides. 

Additional strategies must address individuals’ reluctance to seek professional services, as simply increasing supply does not increase demand. Potential interventions to improve attitudes toward seeking help may include social media campaigns and tailoring interventions to cultural and socio-economic characteristics [26]. 

Future research should focus on understanding the outcomes for the 20% of individuals who have access to professional services but do not find them effective. Additional research could focus on the outcomes for those who decide to do something beneficial on their own. Furthermore, given the widespread mental health impact of COVID-19, the projections we found are likely to change as more research examines these outcomes. Future research should also look at other factors, such as economic status, social support, and access to medication, and the role they might play in suicide rates.

Mental health is a public health crisis, and suicide is a global concern. While recent awareness campaigns have sought to reduce stigma and encourage persons with AMI to seek care, access to professional care remains a barrier. Strategies to develop the mental health workforce and reduce geographical and financial barriers may be effective in increasing access and reducing suicide rates. 

## Figures and Tables

**Table 1:** Revised probabilities for increasing awareness of seeking professional help.

Increase in Awareness (%)	p_11_	p_12_	p_13_	p_14_	p_15_	p_16_
0 (current state)	0.3200	0.5360	0.0000	0.1300	0.0140	0.0000
4	0.2911	0.5760	0.0000	0.1189	0.0140	0.0000
8	0.2627	0.6160	0.0000	0.1073	0.0140	0.0000
12	0.2201	0.676	0.0000	0.0899	0.0140	0.0000

**Table 2:** Results of increasing awareness of seeking professional help.

Increase in Awareness (%)	Probability of Eventually Committing Suicide (%)	Suicide Rate Reduction against Current State (%)	Suicide Rate Reduction against Previous Scenario (%)
0 (current state)	6.3901	-	-
4	6.2482	0.1419	0.1419
8	6.1203	0.2698	0.1278
12	6.0037	0.3864	0.1167

**Table 3:** Revised probabilities for increasing access to professional services.

Increase in Access (%)	p_21_	p_22_	p_23_	p_24_	p_25_	P_26_
0 (current state)	0.1240	0.0000	0.8620	0.0000	0.0140	0.0000
4	0.0840	0.0000	0.9020	0.0000	0.0140	0.0000
8	0.0440	0.0000	0.9420	0.0000	0.0140	0.0000
12	0.0040	0.0000	0.9820	0.0000	0.0140	0.0000

**Table 4:** Results of increasing access to professional services.

Increase in Access (%)	Probability of Eventually Committing Suicide (%)	Suicide Rate Reduction against Current State (%)	Suicide Rate Reduction against Previous Scenario (%)
0 (current state)	6.3901	-	-
4	6.2209	0.1692	0.1692
8	6.0637	0.3263	0.1572
12	5.9173	0.4728	0.1464

**Table 5:** Revised probabilities for increasing awareness and access to professional services.

Increase in Both (%)	p_11_	p_12_	p_14_	p_21_	p_23_
0 (current state)	0.3200	0.5360	0.1300	0.1240	0.8620
2	0.3053	0.5560	0.1247	0.1040	0.8820
4	0.2911	0.5760	0.1189	0.0840	0.9020
6	0.2769	0.5960	0.1131	0.0640	0.9220

**Table 6:** Results of increasing awareness and access to professional services.

Increase in Access (%)	Probability of Eventually Committing Suicide (%)	Suicide Rate Reduction against Current State (%)	Suicide Rate Reduction against Previous Scenario (%)
0 (current state)	6.3901	-	-
2	6.2311	0.1692	0.1692
4	6.0807	0.3263	0.1572
6	5.9373	0.4728	0.1464

## Data Availability

The data that support the findings of this study are available from the corresponding author upon reasonable request.
